# Causal contributors to tissue stiffness and clinical relevance in urology

**DOI:** 10.1038/s42003-021-02539-7

**Published:** 2021-08-26

**Authors:** Laura Martinez-Vidal, Valentina Murdica, Chiara Venegoni, Filippo Pederzoli, Marco Bandini, Andrea Necchi, Andrea Salonia, Massimo Alfano

**Affiliations:** 1 Vita-Salute San Raffaele University, Milan, Italy; 2 Division of Experimental Oncology/Unit of Urology, URI, IRCCS San Raffaele Hospital, Milan, Italy

**Keywords:** Urological manifestations, Cancer microenvironment, Urological cancer, Extracellular matrix

## Abstract

Mechanomedicine is an emerging field focused on characterizing mechanical changes in cells and tissues coupled with a specific disease. Understanding the mechanical cues that drive disease progression, and whether tissue stiffening can precede disease development, is crucial in order to define new mechanical biomarkers to improve and develop diagnostic and prognostic tools. Classically known stromal regulators, such as fibroblasts, and more recently acknowledged factors such as the microbiome and extracellular vesicles, play a crucial role in modifications to the stroma and extracellular matrix (ECM). These modifications ultimately lead to an alteration of the mechanical properties (stiffness) of the tissue, contributing to disease onset and progression. We describe here classic and emerging mediators of ECM remodeling, and discuss state-of-the-art studies characterizing mechanical fingerprints of urological diseases, showing a general trend between increased tissue stiffness and severity of disease. Finally, we point to the clinical potential of tissue stiffness as a diagnostic and prognostic factor in the urological field, as well as a possible target for new innovative drugs.

## Abstract (web summary)

In this Review, Laura Martinez-Vidal et al. summarize current knowledge of factors affecting extracellular matrix (ECM) remodeling in the context of pathological mechanical changes to tissue properties. They focus on the importance of research on tissue stiffness to the development of diagnostic tools and therapies to treat urological disease.

## Introduction

**Fig. 1:**
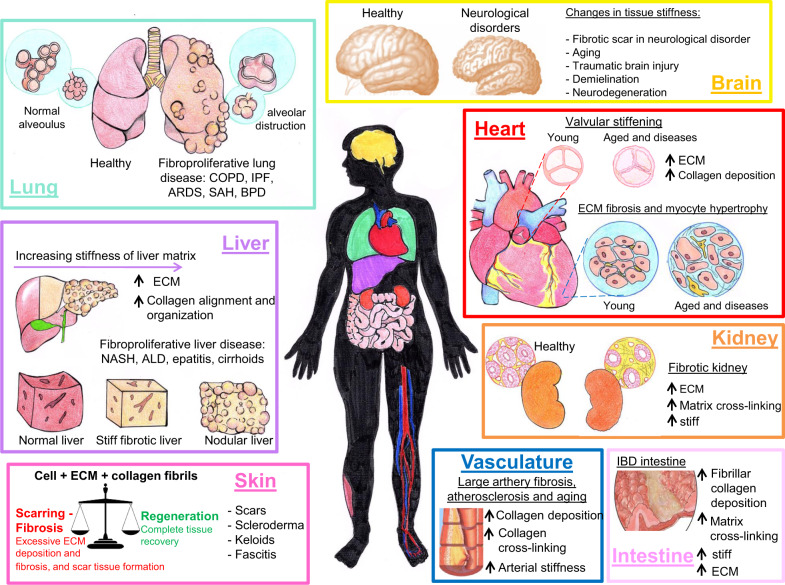
Schematic overview of the major diseases in which tissue stiffness is altered. The hallmark of fibrosis is excessive extracellular matrix (ECM) synthesis and deposition that improve liver matrix remodeling and stiffening. In addition to change the amount of collagen deposited in the ECM, the alignment of the collagen fibrils is also contributing significantly to the alteration of the stiffness of tissues matrix. Increased matrix stiffness is not only a pathological consequence of fibrosis in traditional view but also recognized as a key driver in the pathological progression of fibrosis and cancer. Moreover, it is very likely that significant changes in cell and tissue mechanics contribute to age-related cognitive decline and deficits in memory formation which are accelerated and magnified in neurodegenerative states, such as Alzheimer’s and Parkinson’s disease. COPD chronic obstructive pulmonary disease, IPF idiopathic pulmonary fibrosis, ARDS acute respiratory distress syndrome, SAH pulmonary subarachnoid hemorrhage, BPD bronchopulmonary dysplasia, IBD inflammatory bowel disease, NASH non-alcoholic steatohepatitis, ALD alcoholic liver disease.

The concept that malignancies have an increased consistency and rigidity compared to the surrounding healthy parenchyma is well known in medicine, as palpation of solid masses during physical examination has been the mainstay of tumor diagnosis for centuries. While this example represents an old-fashioned and unaware use of the mechanical properties of tissues for clinical purposes, Mechanics is emerging as a pivotal hallmark for several diseases and pathologies. The extracellular matrix (ECM) is a highly dynamic matrix that, far from being a passive filler, plays important regulatory functions in health and disease. Over the years, it has become clear that the ECM influences cell behavior not only by means of its chemical composition but also through its physical and mechanical properties^1,2^. Remarkably, single cancer cells seem to be softer and more deformable than their normal counterparts, probably to favor their movement and progression within the tumor microenvironment and beyond^3^. Indeed, mechanomedicine is an emerging multidisciplinary field focused on understanding how physical forces and cell and tissue mechanics regulate cell behavior, development, and tissue organization in physiological and pathological conditions^4^. Mechanomedicine, at the intersection between biology, medicine, physics, engineering, and material science, studies how specific diseases have particular mechanical fingerprints^5^ (Fig. 1); this is true of cancer, fibrosis, aging, cardiovascular diseases, and chronic diseases as diabetes. Therefore, quantitative cell and tissue mechanobiology potentially offer the possibility to add a new class of disease hallmarks by identifying the changes in their physical properties.

Regarding living tissues, several mechanical parameters are of clinical relevance, among them stiffness is considered one of the most important and best described mechanical parameters of biological tissues in research^6,7^ (Box 1), and thus our review will focus on stiffness. Pathological changes in tissue stiffness can be traced to altered amounts and/or function of its two fundamental constituents: cells (number and/or phenotype) and ECM (deposition and/or degradation). Knowledge of abnormal tissue states and the mechanical changes that occur in disease are important not only for diagnosis but also for tissue engineering and recapitulation of such diseases in in vitro models. These approaches are expected to generate novel therapeutic targets that allow for the restoration of healthy tissue mechanics and mechanotransduction responses^8,9^. Therefore, it is not surprising that mechano-based therapies that target increased tissue stiffness and associated cellular responses in cancer and fibrosis-mediated diseases are emerging clinically^10^. For instance, targeting of the renin-angiotensin system (RAS) by anti-RAS drugs has been shown to increase the anti-angiogenic effects of bevacizumab in liver metastases of colorectal cancer by reducing the ECM deposition and remodeling operated by metastasis-associated fibroblasts^11^.

Herein, we aim to provide a general overview of the mechanobiology related to tissue stiffness, with a particular emphasis on the available data in benign and malignant urological conditions.

Box 1 Mechanics of materials: basic concepts and definitions

- Stiffness (rigidity): most used mechanical parameter for characterization of living tissues. It gives the resistance to undergoing (elastic) deformation in response to the application of a force, the property of being inflexible and hard to distort^216^. A stiff material, by definition, has a high Young’s modulus (i.e., a considerable stress is needed for a minor deformation). The stiffness of a material is represented by the ratio between stress and strain, being the Young’s modulus (E) the constant of proportionality between stress and strain, and one of the most common measures of intrinsic material stiffness. Units of Young’s modulus are force unit per area, being Pascal (Pa) the most widely used.
- Viscoelasticity: time-dependent anelastic behavior of materials. This means that the temporary response to a stimulus is delayed, and there is a loss of energy inside the material. Viscoelastic behavior normally occurs at different time scales (relaxation times) in the same material^217^. Viscoelasticity can be reported by Shear storage modulus (G′) and shear loss modulus (G″).

## Key contributors to a stiffer matrix

The ECM is a general term used to indicate the non-cellular tissue components that form an essential scaffold for cellular constituents. The structure of ECM differs in composition between tissues but is essentially made up of collagen fibers, proteoglycans, and multiadhesive matrix proteins that are secreted by different cell types^12^. The ECM is a dynamic component of tissues, where there is a constant feedback loop between the ECM and the cells within it: while cells produce and remodel the different ECM components, biochemical and biomechanical features of the ECM direct cell phenotypes. This equilibrium of the ECM turnover and crosstalk with the resident cells is crucial for tissue development and homeostasis.

### The cellular determinants of ECM remodeling

**Fig. 2:**
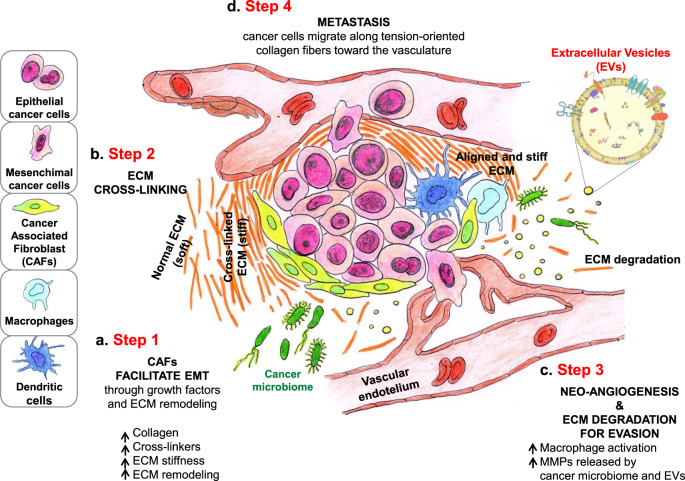
Extracellular matrix and tumor microenvironment. Cell components of the TME (cancer cells, CAFs and TAMs) modulate ECM through different activities. One of these modulations is the topographic reconfiguration of the stroma: ECM anisotropy. By upregulation of LOX, CAFs increase collagen crosslinking, altering ECM topology as well as directionality and mechanical properties. Increased collagen crosslinking induces stiffer microenvironment, which modulates macrophages activation (**a**). Cancer-associated fibroblasts (CAFs) represent the main source of ECM production and remodeling within the TME where they promote neoangiogenesis and EMT (**b**). Tumor-associated macrophages (TAMs), microbiome, and extracellular vesicles (EVs) reshape ECM by secreting MMPs and matrix-associated proteins. MMPs release ECM-attached soluble growth factors and cytokines, which promote neoangiogenesis, contributing to tumor growth and potential metastatic spreading (**c**). Finally, cancer cells migrate along tension-oriented collagen fibers towards the vessels. The alignment of ECM fibers yields a rigid structure that contributes to tumor stiffness and acts as sort of highway for cancer cell migration, leading metastasis (**d**). ECM extracellular matrix, TME tumor microenvironment, CAFs cancer-associated fibroblasts, EMt epithelial–mesenchymal transition, TAMs tumor-associated macrophage, LOX lysyl oxidase enzyme, MMPs matrix metalloproteinases, EVs extracellular vesicles.

Several cell types shape the ECM structure and composition. Among them, fibroblasts are the main producers of ECM components, including structural proteins (e.g., fibrillar and non-fibrillar collagens, elastin), adhesive proteins (e.g., laminin and fibronectin), and proteoglycans^12^. Moreover, they play a pivotal role in overseeing ECM quality and quantity, as they produce different enzymes involved in the maturation and catabolism of collagens, like lysyl oxidase (LOX), which mediates collagen fibers crosslinking (thereby strengthening the ECM), and matrix-degrading enzymes, such as metalloproteinases^13^. Under physiological conditions, fibroblasts are generally quiescent, but they can be activated in response to a plethora of different mechanical and biochemical stimuli^14,15^. Chronic and deregulated fibroblast activation is at the basis of the altered ECM metabolism characterizing several diseases, including cancer. Chronically activated cancer-associated fibroblasts (CAFs) represent the main source of ECM production and remodeling within the tumor microenvironment (Fig. 2a), where they promote neoangiogenesis and epithelial to mesenchymal transformation (EMT)^16–19^.

In addition to fibroblasts, macrophages express genes involved in ECM modulation, like transforming growth factor β induced protein and matrix metalloproteinase 9, which ultimately lead to the release of associated proteins and factors in the stromal environment^20–22^. For instance, a subpopulation of macrophages expressing the lymphatic vessel endothelial hyaluronan receptor 1 (Lyve-1) proteins has been shown to regulate ECM composition in the arterial wall and in the lung^21^. Moreover, macrophages can modulate fibroblast-mediated production of ECM, which contributes in turn to the regulation of fibrosis^23^. It is interesting to note that the mechanical status of the ECM can control the efficacy of TGF-beta activation by reparative fibroblastic cells and the release of traction-mediated latent TGF-β1 stored in the ECM^24^. Macrophages also play an important role in ECM remodeling in the tumor microenvironment. Tumor-associated macrophages (TAMs) may contribute to tumor progression by producing pro-inflammatory cytokines that will ultimately lead to apoptosis suppression and proliferation activation^25^. In addition, TAMs shape tumor ECM by secreting Matrix metalloproteases (MMPs) and matrix-associated proteins, and they organize collagen I into fibrillar bundles, as shown in a mouse model of colorectal cancer^18,26^.

### ECM remodeling proteins

LOX is an extracellular enzyme that catalyzes the crosslinking of collagen and elastin through oxidative deamination of lysine residues^27^. Reduced LOX activity in humans has been described in two X-linked recessively inherited disorders (e.g. Menkes disease and occipital horn syndrome)^28,29^, while elevated LOX levels are clinically associated with increased systemic/organ fibrosis^30,31^. In cancer, LOX plays an important role within the tumor microenvironment since early stages of tumorigenesis^32^ (Fig. 2b). By upregulation of LOX, CAFs increase collagen crosslinking, altering ECM topology, directionality and mechanical properties, ultimately leading to ECM stiffening^33^, which promotes metastasis and infiltration of tumor-supporting immune cells^26^. A positive correlation between LOX expression and cell migration, invasion, EMT, and metastasis has been observed^26,34^. Furthermore, LOX overexpression is an indicator of poor patient prognosis, as seen in several tumors^33,35–38^. Thus, there is potential application of LOX inhibitors in clinical trials to facilitate permeability of drugs and infiltration by tumor-killing immune cells^39^, taking into consideration that LOX inhibitors can only reduce the further crosslinking of collagen fibers, but not restore the already cross-linked ECM.

Matrix metalloproteases (MMPs) are secreted by tumor cells, CAFs, TAMs, and other stromal components (Fig. 2c). MMPs allow for ECM degradation, necessary for cancer cell invasion, and metastasis^18^. These proteases are implicated in almost all steps of metastasis, and an elevated level of MMPs is directly correlated with poor prognosis and a high risk of relapse^40,41^. Furthermore, MMPs release ECM-attached soluble growth factors and cytokines^42^, including vascular endothelial growth factor (VEGF), which will ultimately promote neoangiogenesis, contributing to tumor growth and potential metastatic spreading^43,44^. For example, MMP-9 and MMP-2 cleave TGF-b, promoting tumor invasion, and angiogenesis^45–49^. Werb and colleagues pointed out the role of proteolysis as a mechanism of altering extracellular signaling^50^. For instance, they demonstrated that MMP-2 is a strong contributor to prostate carcinogenesis and that MMP-2 deficiency results in a reduction of immature blood vessel numbers^51^. Likewise, they found that MMP-9 has both pro- and anti-tumorigenic effects, depending on the environment and stage of cancer progression^50,51^. These discoveries fostered new paradigms about the role of MMPs, the microenvironment, inflammation in development and cancer and changed the way in which biomedical researchers view proteolysis, from ECM destruction to extracellular signal transduction^52^. After several clinical trials targeting MMP inhibition led to disappointing results about 20 years ago, probably due to an insufficient knowledge of the MMP interactome and of the pharmacology of the tested compounds, novel ongoing studies are testing MMPs ability to activate prodrugs or facilitate drug delivery^53,54^.

## Tissue mechanics on disease: ECM deposition, topographic reconfiguration of the stroma and cell contribution to tissue stiffness

### ECM stiffness and malignant ECM modifications

The ECM plays a crucial role in the classically defined hallmarks of cancer, from tumor onset, progression, and metastasis^34,35^, and its biochemical and biophysical characteristics undergo constant remodeling^37,55^. During cancer and fibrotic diseases, dysregulated matrix synthesis and remodeling takes place. Cell components of the tumor microenvironment (cancer cells, CAFs and TAMs) modulate ECM through different activities. One of these modulations is the topographic reconfiguration of the stroma: ECM anisotropy (Box 2). The alignment of ECM fibers yields a rigid structure that contributes to tumor stiffness^11,19^. One example is the radial alignment of thick collagen bundles seen at the invasive front in breast cancer^20^. Linearization of collagen fibers has been associated with an increase of tissue stiffness, and both properties are related to the presence of neoplastic tissue and poor prognosis, while non-neoplastic tissue exhibits a more random orientation of fibers and lower tissue stiffness^19,21–23^. The anisotropic organization of biological ECM is not a clinically applied parameter, but several studies have revealed that the significant increase of alignment of collagen fibers (i.e., increase of its anisotropy) has an impact on gene expression, differentiation, proliferation, and migration of cancer cells, with aligned fibers acting as sort of highway for cancer cell migration^21,26^ (Fig. 2d). Interestingly, metastatic breast tumors are characterized by bundles of aligned collagen fibers oriented perpendicular to the tumor interface, highlighting the effect of ECM anisotropy as a strong regulator of directed cell migration^27,28^. Another interesting example of the effect of tissue alignment in cancer invasion regards the pattern of invasion: invading mesenchymal melanoma and sarcoma cells, which are known to typically invade as single cells, can invade collectively when three-dimensional tissue density is high and the activity of MMPs contributes to tissue alignment^56,57^. Moreover, Friedl and coworkers demonstrated that in vivo invading cancer cells preferentially orient along aligned collagen fibers and bundles, vascular structures, and nerves^58–60^, while mesenchymal tumor cells move collectively along these confined trails despite their labile cell–cell junctions^56,61^. Interestingly, anisotropy remodeling of ECM would probably lead to the generation of stiffness gradients, which leads us to introduce the concept of durotaxis. Durotaxis has recently been proposed as a mechanism driving directed migration, which is the process by which cells follow gradients of extracellular mechanical stiffness, from soft to stiff substrate^62^. The concept of durotaxis has also been reported for several cancer cell lines, showing that cells displayed the strongest durotactic migratory response when migrating on the softest regions of stiffness gradients (2–7 kPa), with decreased responsiveness on stiff regions of gradients^63^. On the other side, a stiffer substrate promotes the proliferative capacity of cells and enhances tumor growth^64^. Durotaxis might have an important role in facilitating the forward movement of the invasion front, where tumor cells from the tumor niche move forward following the avenues of the stiffer ECM created by the surrounding CAFs.

Altogether, the increase in ECM deposition and in its alignment facilitates cell proliferation, migration, and tumor cell invasion. As a result, tumors are generally stiffer than the normal tissue, due to the ECM alterations mainly induced by CAFs^18,29^. For example, gradual stiffening of tumor stroma along with increasing tumor aggressiveness has been reported for several cancers, including colorectal and breast malignancies^65^. Another example of the association between ECM remodeling and disease is the fibrotic change in the prostate that results in an increase in the stiffness of the tissue, as a consequence of inflammatory processes^34,66,67^.

Stromal cells contribute to ECM and tissue stiffness and, at the same time, a high stiffness matrix has been suggested to be important for maintaining an invasive phenotype^37^, as well as favoring EMT^68^ facilitating the transition from epithelial and non-motile phenotype to a mesenchymal and motile (invasive) phenotype providing cellular motility^69^. In the case of breast cancer, it has been shown that denser breast tissue is a risk factor for breast cancer development^70,71^. Such increased breast density is a result of increased connective tissue deposition and ECM components, mostly collagen^70^, which is not only increased in deposition but also more oriented, stiffer, and correlated with higher epithelial cell density^71^. Collagen alignment has also been seen to have potential as a prognostic marker for invasive breast carcinoma patients^72,73^. This stiffer stroma increases breast cancer risk by inducing the oncogene *ZNF217*^71^. Furthermore, in breast cancer tumors in vivo initiation of metastasis is promoted by a stiffer microenvironment induced by increased collagen crosslinking^74^. Interestingly, it has been shown that collagen fibril density, which is a surrogate for tissue stiffness, modulates macrophage activation, and cellular functions during tissue repair^38^, and that cytokine secretion is enhanced with an increase of fibril density. In a different study, it was stated that immune cells, such as macrophages are sensitive to the surrounding rigidity: lesions of higher stiffness from human breast cancer biopsies were infiltrated with a higher number of macrophages, resulting in an increased cellular TGF-b signaling^16^.

Box 2 Main concepts on ECM remodeling contributing to stiffness Desmoplasia: increased matrix production with the remodeling of connective tissue structures adjacent to the tumor^218^.

Cancer-associated fibroblasts (CAFs): the main source of ECM production and remodeling within the tumor microenvironment, promoting neoangiogenesis and epithelial to mesenchymal transformation (EMT)^19^.

Lysyl oxidase (LOX): enzyme that converts lysine into high reactive aldehydes forming crosslinking between fibers of collagens and elastin^219^.

Metalloproteases (MMPs): extracellular enzymes that degrade the ECM components^220^.

Tumor-associated macrophages (TAMs): polarized macrophages that suppress antitumor immunity and promote tumor progression.

Anisotropy: reorganization of the topography of the ECM toward linearization of the alignment of ECM fibers.

### Cellular contribution to tissue stiffness: cell stiffness, density, and traction

Not only does ECM contribute to tissue stiffness, but there is an important cellular contribution to the rigidity of their surrounding environment by means of cell stiffness, density, and contractility. As previously mentioned, cancer cells are softer than their non-transformed counterparts^75^. In vitro tests have shown that increasing ECM stiffness increases both tumor cell proliferation and invasiveness^76^, although the softening of cancer cell lines was not associated with the aggressiveness of several solid neoplasia^75,77–79^. Of particular note is the fact that the elasticity of tumor cells now represents one of the markers for the recognition and isolation of circulating tumor cells^80^.

Although it cannot be excluded that a stiffer substrate induces a softening of the tumor cells, it also cannot be excluded that a softening of the tumor cell is an early event associated with the malignant transformation. Indeed, it has been reported that mechanical forces played by the neoplastic cells or cancer-associated fibroblasts in the in situ carcinoma are sufficient to remodel the basal membrane to allow for tissue invasion^81^. On the other hand, it has been reported in vitro that focal adhesion points of the cell with the substrate, resulting in the formation of stress fibers in the cell, mediate cell traction. When cell density is high enough to keep cells close to each other, this cell traction increases the local stiffness, an event that is particularly relevant when high density of tumor fibroblasts or mesenchymal stem cells are seeded on a soft substrate^82^.

Tumor progression is mediated by the ability of tumor cells to invade the tissue layers to reach the vessel and spread in secondary organs. Invasion by in situ carcinoma requires tumor cells to pass the basal membrane to reach the lamina propria located below the epithelial layer. Besides the MMP-mediated remodeling of the ECM, including the ECM composing the basal membrane^83^, physical remodeling of the basal membrane has also been reported to be induced independently of MMPs. The basal membrane can be remodeled by a large protrusion of cancer cells that physically tears and then displaces the basal membrane^84^. On the other hand, CAFs have recently been reported to modify the ECM of the basal membrane independently of MMPs^81^. In the presence of MMP inhibitors, the physical contact between CAFs and the basal membrane was reported to sustain invasion. By applying mechanical forces on the basal membrane, CAFs reduced the density of fibers of intermediate stiffness, creating patches of soft, inhomogeneous material sparsely interspersed with thick fibers. By exerting contractile forces CAFs can soften the basal membrane, thus adding a second mechanism that is proteolysis-independent to the tumor progression of in situ carcinoma. Because this event has not been reported for fibroblasts isolated from the juxta-tumoral tissue, it would be important to unveil the tumor-derived factors (i.e., extracellular vesicles (EVs)) that induce transformation of fibroblast to CAFs and that enhance their mechanical forces.

Increased local stiffness by mechanical forces due to cell traction, proteolysis-independent remodeling of the ECM adds another piece of information regarding tumor progression and why MMP inhibitors have failed in clinical trials.

### The pre-metastatic niche

Tumor cells, together with CAFs, hematopoietic progenitor cells, and TAMs prepare a suitable “soil” for incoming metastasis in distant tissues and organs. This abnormal, tumor growth-favoring microenvironment is the so-called pre-metastatic niche^85^. The ECM is closely related to tumor metastasis, and the tumor-environmental transition from softer tissue to stiff fibrous tissue goes hand in hand with metastasis progression. First, in the primary tumor site, primary tumor cells secrete soluble growth factors (as VEGF-a, TGF-b and tumor necrosis factor alpha) and EVs (see section 4.1) containing miRNAs, integrins, growth factor receptors, and chemoattractants^86,87^. These soluble growth factors and EVs arrive at the target tissue and prime it, as a first step of preparing a suitable microenvironment for metastasis formation^88^. As a second step, bone marrow derived cells, CAFs, myofibroblasts and TAMs colonize the niche, resulting in an altered expression of collagens and MMPs. Finally, tumor cells that underwent EMT arrive at the target tissue as tumor circulating cells, which they colonize to form a second tumor^14,89,90^. Collagen stabilizing enzymes are overexpressed during metastasis and hypoxic conditions, where they play a critical role hydroxylating collagen in response to hypoxia-inducible factors^14^. One example of the priming of the tissue is breast cancer cells that metastasize to lung, where they secrete collagen and change the organization of ECM fibers^13,19^. Colorectal liver metastasis presents high collagen turnover together with collagen isoforms changes, contributing to the generation of a favorable tumor microenvironment^91,92^. On the other hand, metastatic melanoma cells can re-organize the collagen matrix depending on their invasive potential: the higher the invasive potential, the greater the traction force of the cell on the ECM, resulting in a more linearized ECM where cell migration is enhanced^93^.

## Novel mediators of ECM remodeling

### The extracellular vesicles

EVs are small membranous vesicles released into the extracellular environment by virtually all cell types and they act as a “cell-to-cell delivery service” since they contain cell-specific cargo molecules, including proteins, lipids, and nucleic acids^94^. EVs secreted by both cancer cells and cancer‐associated cells, and in particular exosomes (i.e. a particular group of EVs characterized by specific biogenesis and diameter), were shown to transfer several bioactive molecules to other recipient cells, inducing modifications of their environment and facilitating tumor growth, invasion, metastasis and the formation of the pre‐metastatic niches^95–98^.

A recent finding by Huleihel showed that biologic scaffold materials contain active EVs bound by matrix components, which strengthen the general role of EVs as structural and functional components of the ECM^99^. Huleihel reported that EVs are closely associated with the collagen network in biological scaffolds. Moreover, EVs are able to exert autocrine control of directional cell migration in vivo by promoting cell polarization and the assembly of adhesions with the ECM components, such as collagen fibers, via integrin–fibronectin interactions^100^. Similarly, Villasante and coworkers showed that the microenvironmental signal, including the three-dimensionality, composition, and stiffness of the tumor matrix, are all together necessary for recapitulating the properties of exosomes found in the primary tumor^101^.

EVs also contain proteinases, such as MMPs, which assist cell invasion during angiogenesis and cancer progression^102^. Tumor-derived EVs deliver ECM-MMPs inducers which can contribute to matrix degradation in different diseases^103,104^: For instance, EVs derived from ovarian cancer contain high levels of MMP-2, MMP-9, and urokinase-type plasminogen activator proteinases that could degrade the ECM^104^. Similarly, MMP-1, MMP-3, and MMP-13 were reported to promote angiogenesis, tumor proliferation, and invasion also in prostate cancer (PCa)^105^. Likewise, fibroblasts migrating into the collagen matrix were documented to release EVs that carry MMP-9 to act upon the collagen fibrils, which leads to a gradual transformation of the collagen matrix from a laminar to a fibrillar type of architecture^91^. All these phenomena might be, from one side, responsible for the pro-invasive and the pro-angiogenic activities mediated by cancer cells or, on the other side, could also strengthen the interaction of EVs with the matrix itself.

A key step during cancer progression to metastasis is achieving the ability to migrate and invade. It has been demonstrated in bladder cancer that differences in mechanical and complementary activation properties of malignant and non-malignant cell-derived EVs may contribute to increase endothelial disruption as well as tumor growth^92^. Furthermore, endothelial leakiness induced by malignant EVs might be a pre-condition for cancer cell trans-endothelial migration during the metastatic progression^92^.

As tumors progress, they need to recruit new blood vessels to ensure a sufficient supply of oxygen and nutrients; this process requires the release of neovascularization-stimulating factors^93^ and EVs have been implicated in this process. Hypoxia, which impact angiogenesis, tumor progression, and immune tolerance, has been shown to increase EVs release from cancer cells^106,107^. Secondly, the presence of matrix MMPs also allows for the direct modulation of the ECM by EVs in the primary tumor microenvironment and metastatic spread, contributing to ECM degradation to provide spaces for blood vessel recruitment. In renal cell carcinoma, EVs were enriched with azurocidin protein, which is involved in vascular permeabilization^108^. Furthermore, EVs released by bladder-cancer patients were shown to be enriched with EDIL-3, which activated epidermal growth factor receptor signaling in cancer and endothelial cells, promoting their angiogenesis and migration^95^. Similarly, PCa cell-derived EVs contain TGFβ and stimulate the differentiation of bone marrow mesenchymal stem cells into myofibroblasts, which secrete high levels of VEGF-A, HGF, and MMPs, such as MMP-1, -3, and -13, have pro-angiogenic functions and enhanced tumor cell proliferation and progression^105^.

As reported above, another key event during cancer progression is the formation of the pre-metastatic niche. Recently, several studies have reported that the formation of a pre-metastatic niche depends on tumor-derived EVs^109–111^. EVs increase angiogenesis and vascular permeability in the pre-metastatic niche^112–114^ and determine organotropic homing^90^. For instance, exosomes derived from high‐grade bladder cancer educate pre-metastatic niches and facilitate distant metastasis^89^. Similarly, human kidney cancer stem cell-derived CD105-positive microvesicles can also stimulate angiogenesis, thereby promoting the formation of the pre-metastatic niche^112^.

### The microbiota

Among the potential modulators of the ECM properties, the microbiota—i.e., the collection of all microorganisms (bacteria, viruses, and fungi) found in a specific environment—certainly plays a role^115^. Every tissue in the human body has a defined ecological community of bacteria associated with good health (eubiosis), whose disruption—a condition known as dysbiosis—has been central in a variety of diseases, including periodontal pathologies, inflammatory diseases, and cancer. It is worth mentioning that we never refer to individual bacterial communities when talking about the resident human microbiota, but rather to a complex ecosystem (i.e. a biofilm) in which different bacterial entities are embedded in a complex ECM and interact with the host^116^. Bacteria produce a variety of proteases that control biofilm homeostasis and, at the same time, may interact with the host counterpart, mediating host ECM degradation, collagen crosslinking, and cell biomechanical properties^117^. It can be hypothesized that these bacterial enzymes have a role in the physiological and pathological turnover of the host tissue ECM, depending on the status of physiological eubiosis vs. pathological dysbiosis of the microbiota itself. Among these enzymes, elastases, hyaluronidases, alkaline phosphatases, and phospholipase C have been thoroughly studied, especially in the context of infection^118–120^. For instance, bacterial elastases have been shown to degrade the ECM and, by interfering with the turnover of interferon-γ, interact with lymphocyte proliferation and activation^117,121^.

Another class of important ECM-degrading enzymes is represented by bacterial collagenases, which are able to digest the triple helix region of collagens with a broader substrate specificity compared to the eukaryotic counterparts, promoting bacterial spread and host tissue modifications^122–126^. Bacterial collagenases are responsible for tissue degradation, an important step for colonization, invasion, and dissemination in the host tissue, and for the acquisition of nutrients for growth and proliferation. Interestingly, abnormal collagen turnover has been described as a pathological hallmark in many human conditions, such as cancer, arthritis, and atherosclerosis, in which the resident bacterial flora plays a physiopathological role^127,128^. Likewise, bacterial dysbiosis might also contribute to impaired tissue homeostasis and thus onset/progression of fibrosis. Significant reduction of *Clostridia* was reported in human testis with idiopathic germ cell aplasia, which was characterized by fibrosis of the seminiferous tubules^129,130^. Noteworthy is the fact that *Clostridia* releases collagenases that only digest interstitial collagens type I, II, and III^131^. One possible interpretation of this association is that continued collagen production by eukaryotic cells is not counterbalanced by the degradation by collagenases from tissue-resident clostridia, helping to exacerbate the progression of fibrosis.

As described above, LOXs are another class of enzymes important for the mechanobiology of the ECM^132^. In particular, LOX-coding genes have been found in the genome of five major bacterial clades: *Bacteroidetes*, *Actinobacteria*, *Proteobacteria*, *Gemmatimonadetes*, and *Deinococcus-Thermus*^133,134^. It can be hypothesized that bacterial LOX may contribute to the modifications of the matrix stiffness in malignancies in which the resident microbiota has been involved, thus collaborating with CAFs and tumor cells to establish a microenvironment beneficent for cancer growth and dissemination^34,66,135–137^.

Bacterial enzymes are also implicated in post-translational modifications of host proteins^138^. For instance, *Porphyromonas gingivalis* expresses a peptidyl-arginine deaminase able to citrullinate collagen I, modifying the interaction between collagen I and fibroblasts expressing integrin α_11_β_1_, thus contributing to the development and progression of destructive arthritis^139,140^. Despite being plausible to hypothesize a similar role of bacteria-induced post-translational modifications of ECM proteins in the onset and progression of solid malignancies, this has not yet been demonstrated, opening the possibility to further investigations.

So far, we have analyzed how bacteria may contribute to modify the ECM microenvironment to favor the onset or progression of pathological conditions. However, it has been recently reported that the host ECM is also able to influence bacterial behavior and function, in particular the bacterial ability to invade and infect mammalian host cells. By manufacturing hydrogels of varying stiffness seeded with human microvascular endothelial cells, Bastounis et al. found that adhesion of the bacterial pathogen *L. monocytogenes* to host cells increases monotonically with increasing matrix stiffness^141^.

Based on these findings, it seems feasible that the interplay between tissue-resident microbiota and host ECM has a bidirectional relationship of reciprocal influence^135^. During eubiosis, bacterial enzymes may play a role in the physiological turnover of the host ECM, which in turn conveys certain growth signals back to bacteria that maintain a “healthy” and noninvasive bacterial ecosystem. Conversely, in case of dysbiosis, the enzymes produced by the dysbiotic bacteria may contribute to polarize the ECM towards a “malignant” phenotype able to promote the onset and progression of host pathological conditions. At the same time, the “malignant” ECM may contribute to increase the ability of bacteria to invade the host tissue, infect host cells and produce genotoxins and other virulence factors. Future studies are needed to establish the relevance of this hypothesis.

## Organ-specific stiffness

### From micro to macroscale: stiffness in clinical practice

In the next sections, we will review the current knowledge about stiffness in benign and malignant urological diseases. Before proceeding forward, it should be clear to the reader that the different available techniques to study tissue and organ stiffness vary in the resolution of the appreciable modifications (Table 1). Atomic force microscopy (AFM)-based measurements provide the finest resolution in the micro-/nano- scale, but they are limited to ex vivo specimens and by a long acquisition time, making its translation into clinical and diagnostic practice difficult. On the other hand, imaging-based techniques, like magnetic resonance elastography (MRE), shear wave elastography (SWE), and real-time elastography, can only provide macroscale maps of tissue stiffness, thus providing an overview of the organ stiffness. How to complement different techniques with different resolutions is still a matter of research, and future studies are needed to define the framework to integrate micro- and macro-scale stiffness data.

**Table 1:** Commonly used techniques for the mechanical characterization of living tissues.

Technique	Concept	Modulus	Sample	Scale	Ref
Atomic force microscopy (AFM)	Atomic-level indentation (nanoindentation) or shear rheology (atomic force microscopy-based rheology)	E (indentation), G′, G″ (shear)	Ex vivo tissue	Microscale, nanoscale	^209–211^
Shear rheometry	Application of small-amplitude oscillatory shear stress and quantification of the resulting strain	G′, G″ (shear, viscoelastic)	Ex vivo tissue	Macroscale	^212,213^
Compressive deformation	Classic stress-strain analysis. Uniaxial stress is applied to compress the material and a relationship is established with the resulting strain	*E* (elastic)	Ex vivo tissue	Macroscale	^214^
Magnetic resonance elastography (MRE)	Magnetic resonance visualization of tissue deformation resulting from the introduction of shear waves into the tissue derived from external vibrations noninvasive, promising for clinical applications	G′, G″ (shear, viscoelastic)	In vivo tissue,	Macroscale	^215,216^
Real time elastography (RTE)	Sonography-based noninvasive method. It uses conventional ultrasound probes to compare echo signals before and after slight compression	E (elastic)	In vivo tissue	Macroscale	^217^
Shear wave elatography (SWE)	External acoustic force pulses are used to generate shear waves which propagate perpendicular to the ultrasound beam, causing transient displacements that result in an image of the distribution of the shear-wave velocities	Shear wave speed (SWS), that can be converted into E and G	In vivo tissue	Macroscale	^218^

Adapted from Guimarães et al. ^8^.

### Tissue stiffness—kidney

Histology of the kidney affects its mechanical properties, particularly the amount of fibrosis in the parenchyma^142^. Pathological findings of glomerulosclerosis and tubulointerstitial fibrosis obtained through biopsies are associated with poor prognosis in renal diseases, and with lower renal parenchymal elasticity^143^. Renal biopsy, although being the gold standard for assessing fibrosis with histological techniques, is an invasive procedure that can cause complications. In order to develop early diagnostic tools, there is huge interest in developing noninvasive methods to accurately evaluate nephropathy.

SWE provides quantitative information of the average tissue elasticity of the whole organ, based on the assumption that the structure of benign tissue differs from that of cancerous tissue. Therefore, SWE can be used to compare the stiffness of benign and malignant tissue, and establish a threshold of elasticity, i.e., of Young’s modulus, from which the tissue is considered malignant. In patients with chronic kidney disease the renal parenchymal stiffness was measured by SWE, and the obtained Young’s modulus was correlated with serum creatinine, urea levels, and the estimated glomerular filtration rate (eGFR). A positive correlation between stiffness and age, serum creatinine, and urea was observed, while the eGFR level had the opposite trend and was negatively correlated with stiffness^142^. The same study also established that a value of tissue stiffness higher than 4.31 kPa corresponded to a diseased kidney (Table 2), an information that can be used to develop early noninvasive diagnostic tools. Of interest is also the fact that a correlation has been reported between renal stiffness and degree of renal fibrosis^144,145^, with patients suffering from chronic kidney disease exhibiting a stiffer renal parenchymal than non-chronic kidney disease patients^146^. Renal elasticity is therefore a potential predictor of chronic kidney disease^143^. In fact, patients with later stages of chronic kidney disease had stiffer renal cortex, with stiffness increasing progressively from stage 3 to 5 of the disease. Interestingly, a worsening of renal stiffness was associated with proteinuria, which is characterized by the infiltration of inflammatory cells into the renal interstitium and the replacement of the tubulointerstitium with fibrous scar^147^. The worsening of renal elasticity was associated with rapid renal deterioration in these patients, suggesting that proteinuria could be an indicator of renal elasticity and early renal fibrosis. Similar results were observed when comparing renal stiffness values obtained by MRE, which differentiated patients with CKD (5.10 kPa) from those with normally functioning kidneys (4.35 kPa). The mean stiffness in patients with CKD significantly increased from stage 1 to stage 4^148^. At stage 5 CKD (kidney failure) the renal stiffness decreased, likely due to renal hypoperfusion that can mask fibrosis^148,149^. Furthermore, MRE was also evaluated for assessing the chronic renal allograft dysfunction. The skewness of MRE corticomedullary stiffness was sensitive to changes in chronic allograft dysfunction, and the MRE cortical and corticomedullary mean stiffness appears to be a predictor of graft loss/relist; a cutoff of 2.48 and 3.29 kPa for no progressive decline was identified on cortical and corticomedullary stiffness, respectively^150^. In a preclinical model of atherosclerotic renal artery stenosis, MRE was also used to monitor decreased medullary stiffness in response to low-energy shockwave therapy^151^.

**Table 2:** Mechanical moduli of human urological tissues.

Tissue	Technique	Modulus	Modulus value (condition)	Ref
Kidney				
	SWE	YM	4.31 kPa (healthy)	^142^
	RTE		75.1 ± 37.8 (non-CKD) 72.9 ± 37.6 (CKD stage 3a) 59.3 ± 40.3 (CKD stage 3b) 48.3 ± 33.8 (CKD stage 4) 36.6 ± 33.0 (CKD stage 5)	^143^
	MRE	YM	4.35 kPa (normal functioning kidneys) 4.86 kPa (cutoff value) 5.10 kPa (CKD patients)	^148^
	MRE	YM -Cortico-medullary YM -Cortical	3.24 kPa (functional allografts) 3.73 kPa (chronic dysfunction allografts) 3.29 kPa (no progressive decline) 4.82 kPa (graft loss/relist) 2.43 kPa (functional allografts) 2.84 kPa (chronic dysfunction allografts) 2.48 kPa (no progressive decline) 3.67 kPa (graft loss/relist)	^150^
	MRE	SWS	1.86 m/s (IgA neprophaty) 2.05 m/s (cutoff value) 2.34 m/s (healthy)	^152^
Prostate				
	SWE	YM	42 kPa (cutoff value healthy)	^177,180^
	SWE	YM	144.85 kPa (cutoff value for recurrence after radical prostatectomy)	^182^
	SWE	YM	31.79 ± 16.17 kPa (benign) 114.96 ± 85.25 kPa (malignant)	^177^
	SWE	YM	95 ± 28.5 kPa (Gleason score 6) 163 ± 63 kPa (Gleason score 7)	^219^
	SWE	YM	91.6 kPa (Gleason score 6) 102.3 kPa (Gleason score 7) 131.8 kPa (Gleason score 8)	^220^
	SWE	YM	32.7 ± 19.4 kPa (Gleason score 6) 55.4 ± 48.5 kPa (Gleason score 7) 57.3 ± 39.4 kPa (Gleason score 8) 88.2 ± 64.2 kPa (Gleason score 9)	^179^
	MRI with tomoelastograhy	SWS	2.8 ± 0.4 m/s (peripheral zone) 2.8 ± 0.3 m/s (transition zone) 3.1 ± 0.6 m/s (PCa)	^185^
Bladder				
	Rheology	Storage modulus Loss modulus	0.052–0.085 Mpa (healthy) 0.019–0.043 Mpa (healthy)	^165^
Testis				
	SWE	Velocity stiffness	0.76 m/s (normal) 0.79 m/s (testicular microlithiasis) 1.92 m/s (testicular cancer)	^188^
	SWE MRI	Velocity stiffness Diffusion values	0.77 m/s (normal) 0.78 m/s (testicular microlithiasis) 1.95 m/s (testicular cancer) 0.929 × 10^−3^ mm^2^ s^−1^ (normal) 0.978 × 10^−3^ mm^2^ s^−1^ (testicular microlithiasis) 0.743 × 10^−3^ mm^2^ s^−1^ (testicular cancer)	^189^
	SWE	YM	4.5 kPa (cutoff value for semen parameters improvement after surgery)	^190^
	SWVV		1.465 m/s (cutoff normal/oligozoospermia) 1.328 m/s (cutoff normal/azoospermia)	^192^
	SWE	YM	2.50 ± 0.49 kPa (Varicocele grade I) 2.59 ± 0.81 kPa (Varicocele grade II) 2.80 ± 0.72 kPa (Varicocele grade III)	^191^

*CKD* chronic kidney disease, *RTE* real-time elastography (uses arbitrary units), *YM* Young’s modulus, *SWS* shear-wave speed, *SWVV* shear wave velocity values, *MRI* magnetic resonance imaging diffusion values.

Besides MRE, quantitative magnetic resonance imaging (MRI) using tomoelastography has been used to assess renal parenchyma softening in IgA neprophaty (shear wave speed 1.86 m/s vs. 2.34 m/s in age-matched healthy volunteers)^152^. The same authors also stated that a cutoff value of 2.05 m/s was used with a sensitivity and specificity of 81% and 100%, respectively, and identified a positive correlation of shear wave speed and eGFR, still supporting that renal parenchyma softening occurs when kidney perfusion is decreased^152^.

Furthermore, renal elasticity is also a potential predictor for renal transplant outcome^153–157^, as well as an indicator for diabetic nephropathy^146^. Although tissue biopsy is the gold standard for assessing renal fibrosis, this procedure is invasive and prone to sampling error. Imaging techniques, such as MRE, MRI, and SWE constitute promising noninvasive techniques to monitor mechanical changes associated with renal function.

### Kidney specific mechanisms for increased stiffness

Kidney stiffness is associated with kidney fibrosis, age, proteinuria, and poor prognosis. Fibroblasts play a major role in kidney fibrosis, and an important accumulation of fibroblast was reported in the kidney, with 15% of fibroblasts in renal fibrosis originating from bone marrow, 36% from local tubular epithelial cells via EMT and the remaining 50% from proliferating resident fibroblasts^158,159^. Furthermore, Activin A has been reported as a relevant stimuli which induced the expression of fibronectin and collagen I in renal interstitial fibroblasts^19,160^. EMT is crucial for organ fibrosis, i.e., deposition of collagens, elastin, tenascin, and additional matrix proteins. Fibroblast-specific protein 1 (FSP1) is a very interesting protein found in the EMT proteome, whose expression correlates with EMT during kidney fibrosis^161^. Inhibition of FSP1 attenuates fibrosis and collagen deposition, and preliminary studies suggest that FSP1 may sequester p53 from the APC ubiquitination pathway, resulting in an increase of β-catenin levels, and thus, facilitating EMT phenotype.

### Tissue stiffness—bladder

The mechanical properties of the bladder are essential for its function: it has to adapt and stretch to the urine volume it contains and undergo high elastic extension. Although alterations of the mechanical properties of the bladder result in a dysfunction of its physiological role, the mechanical properties of bladder tumors have been scarcely studied. Nevertheless, most benign bladder pathologies are associated with an increase of ECM-fibrosis and may progress from the formation of stiffer matrix to a more compliant structure^162^. Such mechanical information is very important and relevant for several purposes, as is the development of innovative diagnostic tools, computational models, improvement of surgical devices, and surgical trainers.

Already in 1994, bladder wall elasticity was classified as a physiological biomechanical characteristic, susceptible to change with the development of different pathologies^163^. In this study, they measured the ratio of connective tissue to smooth muscle in patients with bladder pathology and compared them to normal bladders. They found that such ratios were increased compared to normal tissues, indicating that in the dysfunctional bladder there is a higher proportion of connective tissue compared to smooth muscle tissue. Although no mechanical tests were performed, they directly correlated these observations with a loss of elasticity in the bladder wall. Interestingly, later published studies have suggested to use the ratio of fibrous connective tissue to smooth muscle tissue measured by SWE as a parameter for the diagnosis of early fibrotic changes^164^.

The viscoelastic properties of human bladder tumors at the macroscale have been tested by different techniques. In one study^165^, researchers analyzed ten bladder human tumors by dynamic mechanical analysis. Samples were collected from patients by transurethral resection procedures, and they applied a load to the tumor with increasing frequencies, up to 30 Hz, calculating both the storage and loss modulus (Table 2). These macroscopic measurements were previously established on porcine bladder by the same group^166^. This analysis quantifies the frequency-dependent viscoelastic properties on the macroscale which is rheometer, getting two different mechanical parameters for each tumor: storage modulus, ranging between 0.052 and 0.085 MPa; and loss modulus ranging between 0.019 and 0.043 MPa.

Efforts are being made to develop new techniques to monitor changes in bladder wall mechanical properties in a noninvasive way^167^. Aiming to develop noninvasive diagnostic tests for lower urinary tract disorders (LUTS), one study compared three quantifiable ultrasound methods (high-frequency ultrasound, SWE, and duplex doppler) to measure the biomechanics of the bladder wall in healthy individuals, in order to establish baselines and reference points for future research. Such quantitative noninvasive diagnostic tool would allow to detect bladder wall changes and decreased wall function before obvious fibrotic changes develop^164^. They observed that bladder wall pathology affects the structure and thickness of the bladder wall layers; and the thickness of the bladder and detrusor layer increased with age. The increase in thickness with age could be related to an increased interstitial collagen deposition or to hypertrophy of the detrusor.

Mechanical properties of bladder cancer have been studied at different scales. At a cellular level, also mechanical properties are of relevance, and there is increasing evidence that the mechanics of cells can be used as a marker for pathology (metastatic potential, differentiation degree, etc.). Bladder cancer cells with different metastatic potential have been characterized^168^, showing that cell lines with higher aggressiveness have a lower Young’s modulus than lower grade cancer cells. These observations have been reported by several studies^75,169–172^, showing that cancer cells are more elastic than their benign counterparts. This increase in elasticity (indicated by a decrease in the cell’s Young’s modulus) means higher deformability of cancer cells, which could have implications for facilitating intra and extravasation and thus, metastasis. This tendency has been observed not only in bladder cancer cells, but also in tissues, such as prostate and breast.

### Bladder specific mechanisms for increased stiffness

Changes in the bladder wall associated with a loss of bladder elasticity and dysfunction could be a consequence of inflammation, loss of urothelium and obstructive or neurogenic etiologies. These processes would lead to hypertrophy of smooth muscle cells, hyperplasia of fibroblasts and the deposition of collagen fibers between muscle bundles of detrusor, and bladder wall thickening, and consequently result in fibrosis, scarring and stiffening of the bladder, together with a progressive reduction in bladder capacity^164,173^.

The ECM plays a fundamental role in cancer progression and metastasis. In particular, collagen stiffness has been proposed to promote BCa progression from non-muscle invasive bladder cancer (NMIBC) to muscle invasive bladder cancer (MIBC), specifically six collagen family members located in the ECM-receptor interaction signaling pathway^66,174^.

Furthermore, the expression of these six collagen genes is negatively correlated with a promising prognosis of BCa patients, overall survival rates, and recurrence-free survival. Therefore, these genes could be considered high progression risk factors and be used as independent effective diagnostic and prognostic biomarkers for BCa, suggesting their potential as targets for clinical treatment.

Interestingly, the linearization and topography of stroma have been seen to differ in MIBC patients^13^. Neoplastic ECM was observed to be more dense and compact, and had an increase of linearization of fibers, as similarly observed in on colorectal^175^ and ovarian cancer^176^. Furthermore, there was a loss of tissue morphology and increased vascularization.

### Tissue stiffness—prostate

PCa has a higher cell and vessel density, which is discernibly stiffer than both benign and normal tissues^177^. Given the noninvasive and cost-effective imaging technology of SWE, several studies have been published aiming to evaluate differences in stiffness between PCa and benign tissue based on Young’s modulus^178^ (Table 2). For all studies discussed here, it was reported that the SWE-obtained Young’s modulus of PCa was significantly higher for benign prostate tissue. The stiffness of PCa in the peripheral area, where most PCa starts, gradually increases with the Gleason Score, which is the grade of the tumor: the higher the Gleason score, the stiffer the tissue is^178,179^. The stiffness values corresponding to each Gleason score differ when reported by different studies. For example, the Young’s modulus value to use as a threshold to discriminate benign from malignant prostate tissue has been reported to be 42^180^, 35^181^, or 31 kPa^177^; and a value of 144 kPa has been proposed as a predictor of biochemical recurrence following radical prostatectomy^182^.

It is important to keep in mind that the prostate is a mechanical heterogeneous tissue: its overall elasticity can vary greatly from zone to zone. In addition, there is patient to patient variability in stiffness. Nevertheless, given the fact that SWE allows for the calculation of elasticity ratios between benign and malignant tissue, this technique provides a standardization and results in user-independent imaging of the prostate. Despite inter-variation among the studies, they all agreed on the utility of measuring prostate stiffness by SWE, as this provides additional information for the detection and biopsy guidance of PCa, enabling a substantial reduction in the number of biopsies while ensuring that few peripheral zone adenocarcinomas are missed. SWE can provide information on tissue elasticity, but the combination of this technique with magnetic resonance can improve the detection of PCa^183^ and provide guidance for more targeted biopsies rather than systematic ones, thus increasing the positive rate of PCa in targeted biopsies^177,184^. The biophysical signature of PCa, characterized by increased stiffness, reduced water diffusion, and increased mechanical fluidity of cancer tissue, correlates with increased cell density and fibrous protein accumulation^185^. SWE as well as tomoelastography with diffusion-weighted MRI have great potential for diagnosis, as they can provide quantitative maps of tissue mechanics. With these data, we can conclude that prostate stiffness (obtained by SWE and/or MRI) can be used as a significant marker to enhance the predictive ability of other clinical histopathological factors for PCa detection and diagnosis in clinical practice.

Prostate stiffness seems to play a role in the development of urological syndromes, such as LUTS^67^. LUTS is indicative of benign prostatic hyperplasia and prostatic calculi are closely associated with reduced urinary flow rate and LUTS severity. In general, most calculi are associated with inflammation of the prostate, characterized by lymphocyte and histiocyte infiltration that will feed the inflammatory loop. This chronic inflammation, through the development of fibrosis and calcification will affect urethral stiffness. Prostatic fibrosis and urethral stiffness have been suggested as potential etiological factors of LUTS^186^. In an additional study, periurethral tissue stiffness from radical prostatectomies were mechanically tested by uniaxial load-unload, showing that the periurethral tissues from patients with LUTS were significantly stiffer and had higher collagen content compared to the periurethral tissue of patients without LUTS^181^. Another group suggested that prostate inflammation can induce fibrotic changes in periurethral prostatic tissues, resulting in increased urethral stiffness and LUTS^187^. Diabetes also seems to induce inflammatory changes that can be associated with the development of prostatic fibrosis^186^.

### Prostate-specific mechanisms for increased stiffness

High periurethral stiffness has been correlated with worse urinary symptoms^67^. An explanation of the correlation between the stiffness of the periurethral tissue and LUTS is that an increase in tissue stiffness can be a consequence of a fibrotic change, reducing elasticity and functionality of the tissue. When fibrosis occurs, tissue stiffness results from myofibroblast accumulation, collagen deposition, and ECM remodeling. It has been suggested that calcification and fibrotic changes in the urethra are a consequence of inflammatory processes around the prostatic urethra^186^. Altogether, these results suggest that prostatic fibrosis increases urethral stiffness, resulting in decreased urethral flexibility that compromises the function of prostatic urethra during micturition^187^.

### Tissue stiffness—testis

Modulation of the tissue stiffness has also been reported in several pathologies of the testis. The stiffness of testicular cancer can be more than double the stiffness of normal testis, suggesting that increased stiffness could be used as a testicular malignancy marker, and detectable by ultrasound elastography^188,189^. In testicular microlithiasis, a modest increase of tissue stiffness compared to normal testicles was measured by SWE^188^, but no malignant characteristics are measured by SWE or MRI diffusion in this condition (stiffness values reported in Table 2)^189^. Thus, benign testicular lesions can be differentiated from malignant ones both by MRI diffusion and elastography. Testis stiffness has also been investigated in the varicocele, where collagen deposition and interstitial testicular edema have been described^190^. Of interest was the predictive potential of SWE for patients undergoing varicocelectomy, which showed a negative correlation of testis stiffness after surgical intervention and improvement of semen analysis parameters (sperm count and motility)^190–192^, pointing to the potential of using SWE tissue stiffness measurements to predict fertility improvement.

### Testis specific mechanisms for increased stiffness

A possible mechanism for the development of testicular fibrosis and increased tissue stiffness has been described. Inflammation and immunological factors contribute to testicular damage: infertile patients exhibit interstitial immune cell infiltration, loss of germinal epithelium, a thickening of the lamina propria and fibrosis of the seminiferous tubules^193^. Activin A seems to play a fundamental role in regulating inflammatory responses of the testis and the development of testicular fibrosis^159^ and it is widely expressed in the testis under physiological conditions, where it is mainly produced by Sertoli cells. Activin A regulates fibrosis by stimulating fibroblast proliferation and differentiation into myofibroblasts. It also activates the transcription factor SMAD2 by phosphorylating it, promoting the transcription of several ECM genes. *Activin A* expression is increased in human testes with leukocytic infiltrate and impaired spermatogenesis, and its expression correlates with severity of the disease. Furthermore, these patients present increased collagen and fibronectin deposition, and thickening of basement membrane and lamina propria. It is hypothesized that fibroblasts and peritubular cells contribute to testicular fibrosis after inflammation, and activin A is implicated in such a process. Therefore, activin antagonists might have beneficial effects in limiting inflammation and fibrosis to treat testicular disease, as reported for kidney^160^.

## Reconciling the contribution of cellular and microenvironment modifications with macroscale observations

The mechanomedicine research field is a rapidly growing area of medicine, although there are a few limitations that will need to be addressed in the near future:

1. Due to the diversity in the techniques and methodologies of the protocols and procedures used by different groups, there is no standardized methodology to assess tissue and organ mechanics^7,8,194^.
2. Working with non-fixed living cells and tissues in the clinical environment continues to be a challenge for the study of cell and tissue mechanics. When a biopsy is performed, for example, in the bladder through cystoscopy, the amount of tissue is very limited and in most cases the amount collected is required entirely by the pathologists to perform their diagnosis. Furthermore, the standard procedure of fixation that tissues undergo after being biopsied is necessary to maintain all histological information as accurately as possible^195^, but creates difficulties for mechanical tests. It is therefore crucial that noninvasive methodologies, such as SWE, are utilized. Furthermore, there are studies for the detection of bladder cancer measuring the mechanical properties of cells collected from urine^196^.
3. On the other hand, we are aware of the gap between proposed molecular mechanisms and reported stiffness values. We have aimed to describe the molecular mechanisms on a microscale: the increase of ECM deposition, the remodeling and reorganization of its components, anisotropy, the effects of cell traction, contractility, proposed EVs, and the microbiome as novel players regulating the molecular processes that ultimately determine tissue stiffness at the organ scale, the macroscale. While macroscale measurements provide an overview of structural modifications, the underlying events/mediators cannot be elucidated through techniques such as elastrography. AFM, optical and/or magnetic tweezers, as well as microaspiration, each of which provides mechanical information at the microscale, may be useful in unveiling the contributions of several variables leading to the modification of the tissue properties. For this purpose, the development and/or implementation of new innovative in vivo microscale mechanical techniques is of critical importance. Linking the observed macroscale stiffness to microscale contributors might potentially drive the development of early diagnostic tools and, more interestingly, prognostic tools that could allow for the identification of regions at risk for relapse of malignancies, i.e., bladder relapse which occurs in up to 70% of NMIBC^197^.

## Conclusions

Aging, neoplasia, and metabolic diseases can lead to fibrosis and an increase of tissue stiffness^5^. Thus, tissue stiffness is a clinically relevant parameter for diagnosis and prognosis purposes, as we have here shown that alteration of this mechanical parameter is coupled with the progression of diseases and malignancies associated with kidney cancer and fibrosis^143^, PCa^177,178^, bladder cancer^167^, testicular cancer^189^, and infertility related diseases^190–192^. Biochemical, topographical, and mechanical modification of the ECM occurs both at the primary and metastatic site^19,21,26^, but there is also an important cellular contribution to the altered tissue stiffness by means of cell density, contractility, and traction^82,93^. At the same time, there is a crosstalk between ECM and cells, being this regulation not only biochemical but also physical, as it has been seen that a stiffer ECM induces cell proliferation and EMT, and favors the migration of the invasion front of the tumor^68^. There are several factors that regulate and remodel ECM, with CAFs being one of the most important players. Furthermore, there is a MMP dependent and independent modification of the ECM that contributes to tumor invasion^56,57^.

We have previously introduced here the concept of ECM anisotropy. Geometry and topographic reconfiguration of the stroma has been seen to differ in malignancies, such as colorectal^65,175,198^, breast^19,26,27^, urogenital^66,67^, and ovarian^176^ cancer. Although tissue anisotropy has yet to be studied in the urological field, analyzing the geometry of the tissue and its anisotropy could provide additional information that could potentially be used as an indicator of increased tissue stiffness and unbalanced/altered ECM production during disease. We have also proposed novel ECM remodelers: EVs and microbiota. EVs have been recognized as key signaling mediators in regulating the tumor microenvironment by transferring several bioactive molecules involved in reprogramming and microenvironment remodeling, tumor angiogenesis, and metastasis^109–111^. They have emerged as pivotal mediators of intercellular communications in local and distant microenvironments under patho/physiological conditions. EVs contain bioactive materials such as proteins, nucleic acids, lipids, and several MMPs^94^. These metalloproteinases are involved in altering the make-up of EVs either through the shedding of transmembrane proteins or by directly contributing to ECM remodeling. Although the nucleic acid and proteomic contents of EVs have been studied for their roles in the development of various diseases and tissue repair, information regarding the secretion and biological activities of EV-associated matrix-remodeling enzymes and their regulators is only just beginning to emerge. We have also introduced the microbiota as an emerging mediator of ECM remodeling. Every tissue in the human body has a defined microbiota associated with health, and the several bacteria can interact with the host cells, with which they are living in close proximity, through a variety of signals^115^. For example, bacteria may influence host cell behavior by modulating the host ECM by releasing different enzymes towards the tissue, like collagenases^122–126^, LOXs^133,134^, and others^140^. At the same time, it is plausible that changes in the host ECM linked to pathological states, such as tumors, fibrosis, or inflammation, can have an impact on the bacterial species associated with the tissue, leading to the expansion of some of them at the expense of others^141^. Although only a few studies have started to tackle this bidirectional interaction between host ECM and tissue-associated microbiota, future studies will shed light on the relevance of our hypotheses.

Even if measured using very different techniques, it has been reported in the literature that tissue mechanics change when cancer occurs^17–19,37,55,65^, with this change generally being an increase in tissue stiffness. We have discussed that tissue mechanics differ in several urological-related pathologies, affecting different organs and tissues, such as the kidney, bladder, prostate, urethra, and testis. But, what are the mechanisms behind this change in the tissue mechanics? Is there a common mechanism affecting all different organs causing the increase in tissue stiffness? Or does a tissue-specific mechanism increase stiffness differently in each different tissue? Several general mechanisms causing an increase in tissue stiffness have been described: inflammation and infiltration of immune cells^16,26^, remodeling of ECM, an increase in collagen deposition and linearization^19,23,175,176^, and fibrotic change^66,67,164^. In addition, the role of specific mediators has been described in more detail for certain organs, such as Activin A for the kidney and testis^159,199^, and collagen family members of the ECM-receptor interaction signaling pathway in BCa^66,174^.

In the last years, systemic treatments with immunotherapy agents have shown promising results for the treatment of cancers, with the immunotherapy response being associated with a decrease in viable tumor cells and increases of immune content, which causes stromal and fibrosis activation due to effects on immune cell function^200^. As reported for several solid tumors, the stiffness of the liver increased in the case of hepatocellular carcinoma (HCC), from 3.2 to 5 kPa as evaluated by MRE^201^. In HCC it was found that HCC stiffness increases for MRE in patients treated with pembrolizumab, and the increased stiffness was significantly correlated with overall survival, time to progression, and the infiltration of immune cells^201^. Integration of the MRE in the diagnostic algorithm for GU malignancies for which immunotherapy has proven efficacious, such as kidney and bladder cancers, may aid in determining which patients would or would not benefit from immune checkpoint inhibitors, as well as help distinguish pseudo-progressions from bona fide progressions^202,203^.

In the urological field, another well-known example of a mechanomedicine therapy comes from the treatment of Peyronie’s disease (PD). PD is caused by the formation of an abnormal fibrotic plaque in the tunica albuginea of the penis, resulting in a curvature of the penile shaft that may impair sexual intercourse^204^. These fibrotic plaques are routinely evaluated by different ultrasound techniques, including SWE to evaluate tissue elasticity while detecting non-palpable plaques^205^. Caused by an excessive healing response, Peyronie’s plaques are generated due to an increased synthesis of connective tissue and an inhibition of collagenase enzymes^206^. Among the potential treatments for this condition, the intralesional injection of collagenase purified from *Clostridium histolyticum* has been used since 1985, and results in a decrease in penile curvature in about one third of treated patients^207^. This collagenase degrades both collagen type I and II within the PD plaque^208^.

Emerging information from the mechanomedicine field reveals the impact of the mechanical properties of tissues on disease onset and progression. The development of therapeutic strategies targeting tissue stiffness might have important clinical potential, not only because stromal stiffness is a hallmark of cancer that facilitates metastasis but also because it impedes the transport of therapeutic agents, reducing the efficacy of the treatment^26^.

## Supplementary information

Transparent Peer Review File

## References

[1] Hadden M (2020). Mechanically stressed cancer microenvironment: role in pancreatic cancer progression. Biochim. Biophys. Acta Rev. Cancer.

[2] Lu P, Weaver VM, Werb Z (2012). The extracellular matrix: a dynamic niche in cancer progression. J. Cell Biol..

[3] Alibert C, Goud B, Manneville J-B (2017). Are cancer cells really softer than normal cells?. Biol. Cell.

[4] Naruse K (2018). Mechanomedicine. Biophys. Rev..

[5] Janmey PA, Miller RT (2011). Mechanisms of mechanical signaling in development and disease. J. Cell Sci..

[6] Darnell M, Gu L, Mooney D (2018). RNA-seq reveals diverse effects of substrate stiffness on mesenchymal stem cells. Biomaterials.

[7] Huang D (2019). Viscoelasticity in natural tissues and engineered scaffolds for tissue reconstruction. Acta Biomater..

[8] Guimarães CF, Gasperini L, Marques AP, Reis RL (2020). The stiffness of living tissues and its implications for tissue engineering. Nat. Rev. Mater..

[9] Mancini ML, Sonis ST (2014). Mechanisms of cellular fibrosis associated with cancer regimen-related toxicities. Front. Pharm..

[10] Tschumperlin DJ, Lagares D (2020). Mechano-therapeutics: targeting mechanical signaling in fibrosis and tumor stroma. Pharmacol. Therapeutics.

[11] Shen Y (2020). Reduction of liver metastasis stiffness improves response to bevacizumab in metastatic colorectal cancer. Cancer Cell.

[12] Frantz C, Stewart KM, Weaver VM (2010). The extracellular matrix at a glance. J. Cell Sci..

[13] Alfano M (2016). Linearized texture of three-dimensional extracellular matrix is mandatory for bladder cancer cell invasion. Sci. Rep..

[14] Karamanos NK, Theocharis AD, Neill T, Iozzo RV (2019). Matrix modeling and remodeling: A biological interplay regulating tissue homeostasis and diseases. Matrix Biol..

[15] DeLeon-Pennell KY, Barker TH, Lindsey ML (2020). Fibroblasts: the arbiters of extracellular matrix remodeling. Matrix Biol..

[16] Goulet CR (2019). Cancer-associated fibroblasts induce epithelial–mesenchymal transition of bladder cancer cells through paracrine IL-6 signalling. BMC Cancer.

[17] Kalluri R (2016). The biology and function of fibroblasts in cancer. Nat. Rev. Cancer.

[18] Paolillo, M. & Schinelli, S. Extracellular matrix alterations in metastatic processes. *Int. J. Mol. Sci.* **20**, 4947 (2019). 10.3390/ijms20194947 PMC6802000 31591367

[19] Liu T, Zhou L, Li D, Andl T, Zhang Y (2019). Cancer-associated fibroblasts build and secure the tumor microenvironment. Front Cell Dev. Biol..

[20] Etich J (2019). Gene expression profiling of the extracellular matrix signature in macrophages of different activation status: relevance for skin wound healing. Int. J. Mol. Sci..

[21] Lim HY (2018). Hyaluronan receptor LYVE-1-expressing macrophages maintain arterial tone through hyaluronan-mediated regulation of smooth muscle cell collagen. Immunity.

[22] Murray PJ, Wynn TA (2011). Protective and pathogenic functions of macrophage subsets. Nat. Rev. Immunol..

[23] Wynn TA, Barron L (2010). Macrophages: master regulators of inflammation and fibrosis. Semin Liver Dis..

[24] Hinz B (2015). The extracellular matrix and transforming growth factor-β1: tale of a strained relationship. Matrix Biol..

[25] Farajzadeh Valilou S, Keshavarz-Fathi M, Silvestris N, Argentiero A, Rezaei N (2018). The role of inflammatory cytokines and tumor associated macrophages (TAMs) in microenvironment of pancreatic cancer. Cytokine Growth Factor Rev..

[26] Emon B, Bauer J, Jain Y, Jung B, Saif T (2018). Biophysics of tumor microenvironment and cancer metastasis - a mini review. Comput Struct. Biotechnol. J..

[27] Wang, T.-H., Hsia, S.-M. & Shieh, T.-M. Lysyl oxidase and the tumor microenvironment. *Int. J. Mol. Sci.* **18**, 62 (2016). 10.3390/ijms18010062 PMC5297697 28036074

[28] Kaler SG (1994). Occipital horn syndrome and a mild Menkes phenotype associated with splice site mutations at the MNK locus. Nat. Genet..

[29] Royce PM, Camakaris J, Danks DM (1980). Reduced lysyl oxidase activity in skin fibroblasts from patients with Menkes’ syndrome. Biochem. J..

[30] Murawaki Y, Kusakabe Y, Hirayama C (1991). Serum lysyl oxidase activity in chronic liver disease in comparison with serum levels of prolyl hydroxylase and laminin. Hepatology.

[31] Kagan HM (2000). Intra- and extracellular enzymes of collagen biosynthesis as biological and chemical targets in the control of fibrosis. Acta Trop..

[32] Li T (2018). Lysyl oxidase family members in urological tumorigenesis and fibrosis. Oncotarget.

[33] Wei B (2017). Human colorectal cancer progression correlates with LOX-induced ECM stiffening. Int J. Biol. Sci..

[34] Mohan V, Das A, Sagi I (2020). Emerging roles of ECM remodeling processes in cancer. Semin. Cancer Biol..

[35] Cui X (2018). Lysyl oxidase-like 2 is highly expressed in colorectal cancer cells and promotes the development of colorectal cancer. Oncol. Rep..

[36] Nilsson, M. et al. High Lysyl Oxidase (LOX) in the non-malignant prostate epithelium predicts a poor outcome in prostate cancer patient managed by watchful waiting. *PLoS One* **10**, e0140985 (2015). 10.1371/journal.pone.0140985 PMC4621025 26501565

[37] Stewart GD (2008). Analysis of hypoxia-associated gene expression in prostate cancer: lysyl oxidase and glucose transporter-1 expression correlate with Gleason score. Oncol. Rep..

[38] Liu Y (2018). Lysyl oxidase: a colorectal cancer biomarker of lung and hepatic metastasis. Thorac. Cancer.

[39] Ye M (2020). Evolving roles of lysyl oxidase family in tumorigenesis and cancer therapy. Pharm. Ther..

[40] Cho NH (2003). Increased expression of matrix metalloproteinase 9 correlates with poor prognostic variables in renal cell carcinoma. Eur. Urol..

[41] Herszényi L, Hritz I, Lakatos G, Varga MZ, Tulassay Z (2012). The behavior of matrix metalloproteinases and their inhibitors in colorectal cancer. Int J. Mol. Sci..

[42] Gonzalez-Avila G (2019). Matrix metalloproteinases participation in the metastatic process and their diagnostic and therapeutic applications in cancer. Crit. Rev. Oncol. Hematol..

[43] Apte RS, Chen DS, Ferrara N (2019). VEGF in signaling and disease: beyond discovery and development. Cell.

[44] Pupa SM, Ménard S, Forti S, Tagliabue E (2002). New insights into the role of extracellular matrix during tumor onset and progression. J. Cell. Physiol..

[45] Bissell MJ, Hines WC (2011). Why don’t we get more cancer? A proposed role of the microenvironment in restraining cancer progression. Nat. Med..

[46] McCawley LJ, Matrisian LM (2001). Matrix metalloproteinases: they’re not just for matrix anymore!. Curr. Opin. Cell Biol..

[47] Yu Q, Stamenkovic I (2000). Cell surface-localized matrix metalloproteinase-9 proteolytically activates TGF-beta and promotes tumor invasion and angiogenesis. Genes Dev..

[48] Lambert V (2003). MMP-2 and MMP-9 synergize in promoting choroidal neovascularization. FASEB J..

[49] Masson V (2005). Contribution of host MMP-2 and MMP-9 to promote tumor vascularization and invasion of malignant keratinocytes. FASEB J..

[50] Egeblad M, Werb Z (2002). New functions for the matrix metalloproteinases in cancer progression. Nat. Rev. Cancer.

[51] Littlepage LE (2010). Matrix metalloproteinases contribute distinct roles in neuroendocrine prostate carcinogenesis, metastasis, and angiogenesis progression. Cancer Res..

[52] Kessenbrock K, Plaks V, Werb Z (2010). Matrix metalloproteinases: regulators of the tumor microenvironment. Cell.

[53] Vartak DG, Gemeinhart RA (2007). Matrix metalloproteases: underutilized targets for drug delivery. J. Drug Target.

[54] Fields, G. B. The rebirth of matrix metalloproteinase inhibitors: moving beyond the dogma. *Cells* **8**, 984 (2019). 10.3390/cells8090984 PMC6769477 31461880

[55] Eble JA, Niland S (2019). The extracellular matrix in tumor progression and metastasis. Clin. Exp. Metastasis.

[56] Haeger A, Krause M, Wolf K, Friedl P (2014). Cell jamming: collective invasion of mesenchymal tumor cells imposed by tissue confinement. Biochim. Biophys. Acta.

[57] Wolf K (2007). Multi-step pericellular proteolysis controls the transition from individual to collective cancer cell invasion. Nat. Cell Biol..

[58] Ilina, O. et al. Intravital microscopy of collective invasion plasticity in breast cancer. *Dis Model Mech.* **11**, (2018). 10.1242/dmm.034330 PMC6176993 29997220

[59] Weigelin B, Bakker G-J, Friedl P (2012). Intravital third harmonic generation microscopy of collective melanoma cell invasion: Principles of interface guidance and microvesicle dynamics. Intravital.

[60] Wolf K (2013). Physical limits of cell migration: control by ECM space and nuclear deformation and tuning by proteolysis and traction force. J. Cell Biol..

[61] Haeger, A. et al. Collective cancer invasion forms an integrin-dependent radioresistant niche. *J. Exp. Med.* **217**, e20181184 (2020). 10.1084/jem.20181184 PMC7037234 31658985

[62] Shellard A, Mayor R (2021). Durotaxis: the hard path from in vitro to in vivo. Dev. Cell.

[63] DuChez BJ, Doyle AD, Dimitriadis EK, Yamada KM (2019). Durotaxis by human cancer cells. Biophys. J..

[64] Romani P, Valcarcel-Jimenez L, Frezza C, Dupont S (2021). Crosstalk between mechanotransduction and metabolism. Nat. Rev. Mol. Cell Biol..

[65] Zhang J, Reinhart-King CA (2020). Targeting tissue stiffness in metastasis: mechanomedicine improves cancer therapy. Cancer Cell.

[66] Zhu H, Chen H, Wang J, Zhou L, Liu S (2019). Collagen stiffness promoted non-muscle-invasive bladder cancer progression to muscle-invasive bladder cancer. Onco Targets Ther..

[67] Kwon, J. K., Kim, D. K., Lee, J. Y., Kim, J. W. & Cho, K. S. Relationship between lower urinary tract symptoms and prostatic urethral stiffness using strain elastography: initial experiences. *J. Clin. Med.* **8**, 1929 (2019). 10.3390/jcm8111929 PMC6912549 31717594

[68] Noguchi S, Saito A, Nagase T (2018). YAP/TAZ signaling as a molecular link between fibrosis and cancer. Int. J. Mol. Sci..

[69] Mierke CT (2019). The matrix environmental and cell mechanical properties regulate cell migration and contribute to the invasive phenotype of cancer cells. Rep. Prog. Phys..

[70] Barcus CE (2017). Elevated collagen-I augments tumor progressive signals, intravasation and metastasis of prolactin-induced estrogen receptor alpha positive mammary tumor cells. Breast Cancer Res..

[71] Northey JJ (2020). Stiff stroma increases breast cancer risk by inducing the oncogene ZNF217. J. Clin. Investig..

[72] Conklin MW (2018). Collagen alignment as a predictor of recurrence after ductal carcinoma in situ. Cancer Epidemiol. Biomark. Prev..

[73] Esbona K (2018). The presence of cyclooxygenase 2, tumor-associated macrophages, and collagen alignment as prognostic markers for invasive breast carcinoma patients. Am. J. Pathol..

[74] Levental KR (2009). Matrix crosslinking forces tumor progression by enhancing integrin signaling. Cell.

[75] Ramos JR, Pabijan J, Garcia R, Lekka M (2014). The softening of human bladder cancer cells happens at an early stage of the malignancy process. Beilstein J. Nanotechnol..

[76] Alonso-Nocelo M (2018). Matrix stiffness and tumor-associated macrophages modulate epithelial to mesenchymal transition of human adenocarcinoma cells. Biofabrication.

[77] Faria EC (2008). Measurement of elastic properties of prostate cancer cells using AFM. Analyst.

[78] Lekka M (2012). Cancer cell detection in tissue sections using AFM. Arch. Biochem. Biophys..

[79] Zemła J (2018). Atomic force microscopy as a tool for assessing the cellular elasticity and adhesiveness to identify cancer cells and tissues. Semin Cell Dev. Biol..

[80] Osmulski P (2014). Nanomechanical biomarkers of single circulating tumor cells for detection of castration resistant prostate cancer. Prostate.

[81] Glentis A (2017). Cancer-associated fibroblasts induce metalloprotease-independent cancer cell invasion of the basement membrane. Nat. Commun..

[82] Venugopal B, Mogha P, Dhawan J, Majumder A (2018). Cell density overrides the effect of substrate stiffness on human mesenchymal stem cells’ morphology and proliferation. Biomater. Sci..

[83] Linder S, Wiesner C, Himmel M (2011). Degrading devices: invadosomes in proteolytic cell invasion. Annu Rev. Cell Dev. Biol..

[84] Kelley LC (2019). Adaptive F-actin polymerization and localized ATP production drive basement membrane invasion in the absence of MMPs. Dev. Cell.

[85] Høye AM, Erler JT (2016). Structural ECM components in the premetastatic and metastatic niche. Am. J. Physiol. Cell Physiol..

[86] Kusmartsev S, Gabrilovich DI (2006). Effect of tumor-derived cytokines and growth factors on differentiation and immune suppressive features of myeloid cells in cancer. Cancer Metastasis Rev..

[87] Whiteside TL (2016). Tumor-derived exosomes and their role in cancer progression. Adv. Clin. Chem..

[88] Guo Y (2019). Effects of exosomes on pre-metastatic niche formation in tumors. Mol. Cancer.

[89] Yoshida K (2019). Exosomes containing ErbB2/CRK induce vascular growth in premetastatic niches and promote metastasis of bladder cancer. Cancer Sci..

[90] Lu X, Kang Y (2007). Organotropism of breast cancer metastasis. J. Mammary Gland Biol. Neoplasia.

[91] Masci, V. L., Taddei, A. R., Gambellini, G., Giorgi, F. & Fausto, A. M. Microvesicles shed from fibroblasts act as metalloproteinase carriers in a 3-D collagen matrix. *J. Circ. Biomark.* **5**, (2016). 10.1177/1849454416663660 PMC5548308 28936262

[92] Whitehead B (2015). Tumour exosomes display differential mechanical and complement activation properties dependent on malignant state: implications in endothelial leakiness. J. Extracell. Vesicles.

[93] Weis SM, Cheresh DA (2011). Tumor angiogenesis: molecular pathways and therapeutic targets. Nat. Med..

[94] Raposo G, Stoorvogel W (2013). Extracellular vesicles: exosomes, microvesicles, and friends. J. Cell Biol..

[95] Beckham Carla J (2014). Bladder cancer exosomes contain EDIL-3/Del1 and facilitate cancer progression. J. Urol..

[96] Braicu C (2015). Exosomes as divine messengers: are they the Hermes of modern molecular oncology?. Cell Death Differ..

[97] Franzen CA (2015). Urothelial cells undergo epithelial-to-mesenchymal transition after exposure to muscle invasive bladder cancer exosomes. Oncogenesis.

[98] Yang L, Wu X-H, Wang D, Luo C-L, Chen L-X (2013). Bladder cancer cell-derived exosomes inhibit tumor cell apoptosis and induce cell proliferation in vitro. Mol. Med. Rep..

[99] Huleihel L (2016). Matrix-bound nanovesicles within ECM bioscaffolds. Sci. Adv..

[100] Sung, B. H., Ketova, T., Hoshino, D., Zijlstra, A. & Weaver, A. M. Directional cell movement through tissues is controlled by exosome secretion. *Nat. Commun.* **6**, 7164 (2015). 10.1038/ncomms8164 PMC4435734 25968605

[101] Villasante A (2016). Recapitulating the size and cargo of tumor exosomes in a tissue-engineered model. Theranostics.

[102] Arasu UT (2017). Human mesenchymal stem cells secrete hyaluronan-coated extracellular vesicles. Matrix Biol..

[103] D’Souza-Schorey C, Clancy JW (2012). Tumor-derived microvesicles: shedding light on novel microenvironment modulators and prospective cancer biomarkers. Genes Dev..

[104] Graves LE (2004). Proinvasive properties of ovarian cancer ascites-derived membrane vesicles. Cancer Res.

[105] Chowdhury R (2015). Cancer exosomes trigger mesenchymal stem cell differentiation into pro-angiogenic and pro-invasive myofibroblasts. Oncotarget.

[106] Patton MC, Zubair H, Khan MA, Singh S, Singh AP (2020). Hypoxia alters the release and size distribution of extracellular vesicles in pancreatic cancer cells to support their adaptive survival. J. Cell. Biochem..

[107] Mora S, Muniz-Garcia A, Zorzano A (2020). Molecular mechanisms of hypoxia-induced extracellular vesicle release. FASEB J..

[108] Jingushi K (2018). Extracellular vesicles isolated from human renal cell carcinoma tissues disrupt vascular endothelial cell morphology via azurocidin. Int. J. Cancer.

[109] Peinado H, Lavotshkin S, Lyden D (2011). The secreted factors responsible for pre-metastatic niche formation: Old sayings and new thoughts. Semin. Cancer Biol..

[110] Peinado H (2012). Melanoma exosomes educate bone marrow progenitor cells toward a pro-metastatic phenotype through MET. Nat. Med..

[111] Hood JL, San RS, Wickline SA (2011). Exosomes released by melanoma cells prepare sentinel lymph nodes for tumor Metastasis. Cancer Res..

[112] Grange C (2011). Microvesicles released from human renal cancer stem cells stimulate angiogenesis and formation of lung premetastatic niche. Cancer Res.

[113] Horie K (2017). Exosomes expressing carbonic anhydrase 9 promote angiogenesis. Biochem. Biophys. Res. Commun..

[114] Zeng Z (2018). Cancer-derived exosomal miR-25-3p promotes pre-metastatic niche formation by inducing vascular permeability and angiogenesis. Nat. Commun..

[115] Alfano M (2016). The interplay of extracellular matrix and microbiome in urothelial bladder cancer. Nat. Rev. Urol..

[116] Dragoš A, Kovács ÁT (2017). The peculiar functions of the bacterial extracellular matrix. Trends Microbiol..

[117] Vollmer P, Walev I, Rose-John S, Bhakdi S (1996). Novel pathogenic mechanism of microbial metalloproteinases: liberation of membrane-anchored molecules in biologically active form exemplified by studies with the human interleukin-6 receptor. Infect. Immun..

[118] Sokolova O, Vieth M, Naumann M (2013). Protein kinase C isozymes regulate matrix metalloproteinase-1 expression and cell invasion in Helicobacter pylori infection. Gut.

[119] Estaki M, DeCoffe D, Gibson DL (2014). Interplay between intestinal alkaline phosphatase, diet, gut microbes and immunity. World J. Gastroenterol..

[120] Hynes WL, Walton SL (2000). Hyaluronidases of Gram-positive bacteria. FEMS Microbiol Lett..

[121] Horvat RT, Parmely MJ (1988). Pseudomonas aeruginosa alkaline protease degrades human gamma interferon and inhibits its bioactivity. Infect. Immun..

[122] Duarte AS, Correia A, Esteves AC (2016). Bacterial collagenases – a review. Crit. Rev. Microbiol..

[123] Matsushita O, Koide T, Kobayashi R, Nagata K, Okabe A (2001). Substrate recognition by the collagen-binding domain of Clostridium histolyticum class I collagenase. J. Biol. Chem..

[124] Mookhtiar KA, Van Wart HE (1992). Clostridium histolyticum collagenases: a new look at some old enzymes. Matrix Suppl..

[125] Watanabe K (2004). Collagenolytic proteases from bacteria. Appl. Microbiol. Biotechnol..

[126] Gross J (1974). Animal collagenases: specificity of action, and structures of the substrate cleavage site. Biochem. Biophys. Res. Commun..

[127] Rekhter MD (1999). Collagen synthesis in atherosclerosis: too much and not enough. Cardiovasc. Res..

[128] Xu S (2019). The role of collagen in cancer: from bench to bedside. J. Transl. Med..

[129] Alfano M (2018). Testicular microbiome in azoospermic men-first evidence of the impact of an altered microenvironment. Hum. Reprod..

[130] Alfano M (2019). Impaired testicular signaling of vitamin A and vitamin K contributes to the aberrant composition of the extracellular matrix in idiopathic germ cell aplasia. Fertil. Steril..

[131] French MF, Bhown A, Van Wart HE (1992). Identification of Clostridium histolyticum collagenase hyperreactive sites in type I, II, and III collagens: lack of correlation with local triple helical stability. J. Protein Chem..

[132] Amendola, P. G., Reuten, R. & Erler, J. T. Interplay between LOX enzymes and integrins in the tumor microenvironment. *Cancers* **11**, 729 (2019). 10.3390/cancers11050729 PMC6562985 31130685

[133] Grau-Bové, X., Ruiz-Trillo, I. & Rodriguez-Pascual, F. Origin and evolution of lysyl oxidases. *Sci. Rep.* **5**, (2015). 10.1038/srep10568 PMC4448552 26024311

[134] Mäki JM (2009). Lysyl oxidases in mammalian development and certain pathological conditions. Histol. Histopathol..

[135] Pederzoli, F. et al. Sex-specific Alterations in the Urinary and Tissue Microbiome in Therapy-naïve Urothelial Bladder Cancer Patients. Eur Urol Oncol. **3**, 784–788 (2020). 10.1016/j.euo.2020.04.002 32345542

[136] Pederzoli, F. et al. Is There a Detrimental Effect of Antibiotic Therapy in Patients with Muscle-invasive Bladder Cancer Treated with Neoadjuvant Pembrolizumab? Eur Urol. **80**, 319–322 (2021). 10.1016/j.eururo.2021.05.018 34053782

[137] Pederzoli F (2019). Incremental utility of adjuvant chemotherapy in muscle-invasive bladder cancer: quantifying the relapse risk associated with therapeutic effect (Figure presented.). Eur. Urol..

[138] Ribet D, Cossart P (2010). Post-translational modifications in host cells during bacterial infection. FEBS Lett..

[139] Zeltz C, Gullberg D (2014). Post-translational modifications of integrin ligands as pathogenic mechanisms in disease. Matrix Biol..

[140] Maresz KJ (2013). Porphyromonas gingivalis facilitates the development and progression of destructive arthritis through its unique bacterial peptidylarginine deiminase (PAD). PLoS Pathog..

[141] Bastounis EE, Yeh Y-T, Theriot JA (2018). Matrix stiffness modulates infection of endothelial cells by Listeria monocytogenes via expression of cell surface vimentin. MBoC.

[142] Leong SS (2018). Shear wave elastography in the evaluation of renal parenchymal stiffness in patients with chronic kidney disease. Br. J. Radio..

[143] Lin HY-H (2017). Association of renal elasticity and renal function progression in patients with chronic kidney disease evaluated by real-time ultrasound elastography. Sci. Rep..

[144] Cui G (2014). Evaluation of acoustic radiation force impulse imaging for the clinicopathological typing of renal fibrosis. Exp. Ther. Med.

[145] Nakao T (2015). Evaluation of renal allograft fibrosis by transient elastography (Fibro Scan). Transplant. Proc..

[146] Goya C (2015). Acoustic radiation force impulse imaging for evaluation of renal parenchyma elasticity in diabetic nephropathy. AJR Am. J. Roentgenol..

[147] Brunskill NJ (2005). Rapamycin: a new string to the antiproteinuric bow?. J. Am. Soc. Nephrol..

[148] Han JH, Ahn J-H, Kim J-S (2020). Magnetic resonance elastography for evaluation of renal parenchyma in chronic kidney disease: a pilot study. Radio. Med..

[149] Brown RS (2020). The utility of magnetic resonance imaging for noninvasive evaluation of diabetic nephropathy. Nephrol. Dial. Transpl..

[150] Kennedy P (2020). Magnetic resonance elastography vs. point shear wave ultrasound elastography for the assessment of renal allograft dysfunction. Eur. J. Radio..

[151] Zhang X (2018). Magnetic resonance elastography can monitor changes in medullary stiffness in response to treatment in the swine ischemic kidney. MAGMA.

[152] Lang ST (2019). Multiparametric quantitative MRI for the detection of iga nephropathy using tomoelastography, DWI, and BOLD imaging. Investig. Radio..

[153] Gao J (2013). Renal transplant elasticity ultrasound imaging: correlation between normalized strain and renal cortical fibrosis. Ultrasound Med. Biol..

[154] Gao J (2013). Corticomedullary strain ratio: a quantitative marker for assessment of renal allograft cortical fibrosis. J. Ultrasound Med..

[155] Lukenda V (2014). Transient elastography: a new noninvasive diagnostic tool for assessment of chronic allograft nephropathy. Int Urol. Nephrol..

[156] Orlacchio A (2014). Kidney transplant: usefulness of real-time elastography (RTE) in the diagnosis of graft interstitial fibrosis. Ultrasound Med. Biol..

[157] Tatar IG, Teber MA, Ogur T, Kurt A, Hekimoglu B (2014). Real time sonoelastographic evaluation of renal allografts in correlation with clinical prognostic parameters: comparison of linear and convex transducers according to segmental anatomy. Med. Ultrason.

[158] Kalluri R, Neilson EG (2003). Epithelial-mesenchymal transition and its implications for fibrosis. J. Clin. Investig..

[159] Kauerhof AC (2019). Investigation of activin A in inflammatory responses of the testis and its role in the development of testicular fibrosis. Hum. Reprod..

[160] Maeshima A (2014). Follistatin, an activin antagonist, ameliorates renal interstitial fibrosis in a rat model of unilateral ureteral obstruction. Biomed. Res. Int.

[161] Lee JM, Dedhar S, Kalluri R, Thompson EW (2006). The epithelial–mesenchymal transition: new insights in signaling, development, and disease. J. Cell Biol..

[162] Fry CH (2018). Fibrosis and the bladder, implications for function ICI-RS 2017. Neurourol. Urodyn..

[163] Landau EH (1994). Loss of elasticity in dysfunctional bladders: urodynamic and histochemical correlation. J. Urol..

[164] Volikova AI (2019). Structural, biomechanical and hemodynamic assessment of the bladder wall in healthy subjects. Res. Rep. Urol..

[165] Barnes SC (2016). Viscoelastic properties of human bladder tumours. J. Mech. Behav. Biomed. Mater..

[166] Barnes SC, Shepherd DET, Espino DM, Bryan RT (2015). Frequency dependent viscoelastic properties of porcine bladder. J. Mech. Behav. Biomed. Mater..

[167] Nenadic I (2016). Noninvasive evaluation of bladder wall mechanical properties as a function of filling volume: potential application in bladder compliance assessment. PLoS One.

[168] Liu H (2014). Biophysical characterization of bladder cancer cells with different metastatic potential. Cell Biochem. Biophys..

[169] Canetta E (2014). Discrimination of bladder cancer cells from normal urothelial cells with high specificity and sensitivity: combined application of atomic force microscopy and modulated Raman spectroscopy. Acta Biomater..

[170] Lekka M (1999). Elasticity of normal and cancerous human bladder cells studied by scanning force microscopy. Eur. Biophys. J..

[171] Lekka M (2012). Cancer cell recognition–mechanical phenotype. Micron.

[172] Lin H-H (2015). Mechanical phenotype of cancer cells: cell softening and loss of stiffness sensing. Oncotarget.

[173] Uvelius B, Persson L, Mattiasson A (1984). Smooth muscle cell hypertrophy and hyperplasia in the rat detrusor after short-time infravesical outflow obstruction. J. Urol..

[174] Pederzoli F (2020). Targetable gene fusions and aberrations in genitourinary oncology. Nat. Rev. Urol..

[175] Nebuloni M (2016). Insight on colorectal carcinoma infiltration by studying perilesional extracellular matrix. Sci. Rep..

[176] Nadiarnykh O, LaComb RB, Brewer MA, Campagnola PJ (2010). Alterations of the extracellular matrix in ovarian cancer studied by Second Harmonic Generation imaging microscopy. BMC Cancer.

[177] Fu S, Tang Y, Tan S, Zhao Y, Cui L (2020). Diagnostic value of transrectal shear wave elastography for prostate cancer detection in peripheral zone: comparison with magnetic resonance imaging. J. Endourol..

[178] Yang Y, Zhao X, Zhao X, Shi J, Huang Y (2019). Value of shear wave elastography for diagnosis of primary prostate cancer: a systematic review and meta-analysis. Med Ultrason.

[179] Woo S, Kim SY, Cho JY, Kim SH (2014). Shear wave elastography for detection of prostate cancer: a preliminary study. Korean J. Radio..

[180] Rouvière O (2017). Stiffness of benign and malignant prostate tissue measured by shear-wave elastography: a preliminary study. Eur. Radio..

[181] Ma J (2012). Prostatic fibrosis is associated with lower urinary tract symptoms. J. Urol..

[182] Wei C (2019). Prediction of postprostatectomy biochemical recurrence using quantitative ultrasound shear wave elastography imaging. Front. Oncol..

[183] Mousavi SR, Wang H, Hesabgar SM, Scholl TJ, Samani A (2015). A novel shape-similarity-based elastography technique for prostate cancer assessment. Med. Phys..

[184] Hwang SI, Lee HJ (2014). The future perspectives in transrectal prostate ultrasound guided biopsy. Prostate Int.

[185] Asbach P (2020). In vivo quantification of water diffusion, stiffness, and tissue fluidity in benign prostatic hyperplasia and prostate cancer. Investig. Radio..

[186] Han JH (2015). Is periurethral calcification associated with urinary flow rate and symptom severity in men with lower urinary tract symptoms-benign prostatic hyperplasia? A retrospective review. Urology.

[187] Cantiello F (2013). Periurethral fibrosis secondary to prostatic inflammation causing lower urinary tract symptoms: a prospective cohort study. Urology.

[188] Pedersen MR (2017). Comparison of tissue stiffness using shear wave elastography in men with normal testicular tissue, testicular microlithiasis and testicular cancer. Ultrasound Int Open.

[189] Pedersen MR (2019). Elastography and diffusion-weighted MRI in patients with testicular microlithiasis, normal testicular tissue, and testicular cancer: an observational study. Acta Radio..

[190] Abdelwahab K (2017). Role of preoperative testicular shear wave elastography in predicting improvement of semen parameters after varicocelectomy for male patients with primary infertility. Urology.

[191] Jedrzejewski G, Osemlak P, Wieczorek AP, Nachulewicz P (2019). Prognostic values of shear wave elastography in adolescent boys with varicocele. J. Pediatr. Urol..

[192] Yavuz A (2018). Reliability of testicular stiffness quantification using shear wave elastography in predicting male fertility: a preliminary prospective study. Med Ultrason.

[193] Fijak M (2018). Infectious, inflammatory and ‘autoimmune’ male factor infertility: how do rodent models inform clinical practice?. Hum. Reprod. Update.

[194] Garra, B. S. CliniCal Elasticity Estimation and Imaging: applications and standards. in *Tissue Elasticity Imaging* (eds Alam, S. K. & Garra, B. S.) 1–19 (Elsevier, 2020). 10.1016/B978-0-12-809662-8.00001-2.

[195] Zimmon DS (2017). Endoscopic multiple biopsy and rapid diagnosis by in situ fixation and histopathologic processing. Gastrointest. Endosc..

[196] Sokolov I (2018). Noninvasive diagnostic imaging using machine-learning analysis of nanoresolution images of cell surfaces: Detection of bladder cancer. Proc. Natl Acad. Sci. USA.

[197] Kaufman DS, Shipley WU, Feldman AS (2009). Bladder cancer. Lancet.

[198] Despotović SZ (2020). Altered organization of collagen fibers in the uninvolved human colon mucosa 10 cm and 20 cm away from the malignant tumor. Sci. Rep..

[199] Maeshima A (2014). Follistatin, an activin antagonist, ameliorates renal interstitial fibrosis in a rat model of unilateral ureteral obstruction. BioMed. Res. Int..

[200] Jiang H, Hegde S, DeNardo DG (2017). Tumor-associated fibrosis as a regulator of tumor immunity and response to immunotherapy. Cancer Immunol. Immunother..

[201] Qayyum A (2019). Immunotherapy response evaluation with magnetic resonance elastography (MRE) in advanced HCC. J. Immunother. Cancer.

[202] Hygino da Cruz LC, Rodriguez I, Domingues RC, Gasparetto EL, Sorensen AG (2011). Pseudoprogression and pseudoresponse: imaging challenges in the assessment of posttreatment glioma. AJNR Am. J. Neuroradiol..

[203] Pederzoli, F. et al. Neoadjuvant chemotherapy or immunotherapy for clinical T2N0 muscle-invasive bladder cancer: time to change the paradigm? *Eur. Urol. Oncol.* (2020). 10.1016/j.euo.2020.07.006 32847746

[204] Salonia, A. et al. EAU guidelines on sexual and reproductive health 2020. in *European Association of Urology Guidelines*. 2020 edn. Vol. presented at the EAU Annual Congress Amsterdam 2020. (European Association of Urology Guidelines Office, 2020).

[205] Chen JY, Hockenberry MS, Lipshultz LI (2018). Objective assessments of Peyronie’s disease. Sex. Med. Rev..

[206] Al-Thakafi S, Al-Hathal N (2016). Peyronie’s disease: a literature review on epidemiology, genetics, pathophysiology, diagnosis and work-up. Transl. Androl. Urol..

[207] Gelbard M (2013). Clinical efficacy, safety and tolerability of collagenase clostridium histolyticum for the treatment of peyronie disease in 2 large double-blind, randomized, placebo controlled phase 3 studies. J. Urol..

[208] Capoccia E, Levine LA (2018). Contemporary review of Peyronie’s disease treatment. Curr. Urol. Rep..

[209] Crichton ML (2011). The viscoelastic, hyperelastic and scale dependent behaviour of freshly excised individual skin layers. Biomaterials.

[210] Cross SE, Jin Y-S, Rao J, Gimzewski JK (2007). Nanomechanical analysis of cells from cancer patients. Nat. Nanotech.

[211] Uriarte JJ (2016). Early impairment of lung mechanics in a murine model of marfan syndrome. PLOS One.

[212] Schachar RA, Chan RW, Fu M (2011). Viscoelastic properties of fresh human lenses under 40 years of age: implications for the aetiology of presbyopia. Br. J. Ophthalmol..

[213] Yoo L, Gupta V, Lee C, Kavehpore P, Demer JL (2011). Viscoelastic properties of bovine orbital connective tissue and fat: constitutive models. Biomech. Model Mechanobiol..

[214] Barak MM, Black MA (2018). A novel use of 3D printing model demonstrates the effects of deteriorated trabecular bone structure on bone stiffness and strength. J. Mech. Behav. Biomed. Mater..

[215] Shi Y, Glaser KJ, Venkatesh SK, Ben-Abraham EI, Ehman RL (2015). Feasibility of using 3D MR elastography to determine pancreatic stiffness in healthy volunteers. J. Magn. Reson Imaging.

[216] Murphy, M. C. et al. Measuring the characteristic topography of brain stiffness with magnetic resonance elastography. *PLoS One* **8**, e81668 (2013). 10.1371/journal.pone.0081668 PMC3847077 24312570

[217] Friedrich-Rust M (2007). Real-time elastography for noninvasive assessment of liver fibrosis in chronic viral hepatitis. AJR Am. J. Roentgenol..

[218] Taljanovic MS (2017). Shear-wave elastography: basic physics and musculoskeletal applications. Radiographics.

[219] Ahmad S, Cao R, Varghese T, Bidaut L, Nabi G (2013). Transrectal quantitative shear wave elastography in the detection and characterisation of prostate cancer. Surg. Endosc..

[220] Wei C (2018). Performance characteristics of transrectal shear wave elastography imaging in the evaluation of clinically localized prostate cancer: a prospective study. J. Urol..

